# Commercially Available Flavonols Are Better SARS-CoV-2 Inhibitors than Isoflavone and Flavones

**DOI:** 10.3390/v14071458

**Published:** 2022-06-30

**Authors:** Otávio Augusto Chaves, Natalia Fintelman-Rodrigues, Xuanting Wang, Carolina Q. Sacramento, Jairo R. Temerozo, André C. Ferreira, Mayara Mattos, Filipe Pereira-Dutra, Patrícia T. Bozza, Hugo Caire Castro-Faria-Neto, James J. Russo, Jingyue Ju, Thiago Moreno L. Souza

**Affiliations:** 1 Laboratory of Immunopharmacology, Oswaldo Cruz Institute (IOC), Oswaldo Cruz Foundation (Fiocruz), Rio de Janeiro 21040-900, Brazil; nataliafintelman@gmail.com (N.F.-R.); carol.qsacramento@gmail.com (C.Q.S.); andre.bio2009@gmail.com (A.C.F.); maymattos03@gmail.com (M.M.); filipe.spd@gmail.com (F.P.-D.); pbozza@gmail.com (P.T.B.); hugocfneto@gmail.com (H.C.C.-F.-N.); 2 National Institute for Science and Technology on Innovation on Neglected Diseases (INCT/IDN), Center for Technological Development in Health (CDTS), Oswaldo Cruz Foundation (Fiocruz), Rio de Janeiro 21040-900, Brazil; 3 Center for Genome Technology and Biomolecular Engineering, Columbia University, New York, NY 10027, USA; xw2467@columbia.edu (X.W.); jjr4@columbia.edu (J.J.R.); dj222@columbia.edu (J.J.); 4 Department of Chemical Engineering, Columbia University, New York, NY 10027, USA; 5 Laboratory on Thymus Research, Oswaldo Cruz Institute (IOC), Oswaldo Cruz Foundation (Fiocruz), Rio de Janeiro 21040-900, Brazil; jairo.jrt@gmail.com; 6 National Institute for Science and Technology on Neuroimmunomodulation (INCT/NIM), Oswaldo Cruz Institute (IOC), Oswaldo Cruz Foundation (Fiocruz), Rio de Janeiro 21040-900, Brazil; 7 Preclinical Research Laboratory, Universidade Iguaçu—UNIG, Nova Iguaçu 26260-045, Brazil; 8 Department of Molecular Pharmacology and Therapeutics, Columbia University, New York, NY 10032, USA

**Keywords:** SARS-CoV-2, COVID-19, natural product, flavonoids, molecular docking, calu-3 cells, exonuclease, protease

## Abstract

Despite the fast development of vaccines, severe acute respiratory syndrome coronavirus 2 (SARS-CoV-2) is still circulating and generating variants of concern (VoC) that escape the humoral immune response. In this context, the search for anti-SARS-CoV-2 compounds is still essential. A class of natural polyphenols known as flavonoids, frequently available in fruits and vegetables, is widely explored in the treatment of different diseases and used as a scaffold for the design of novel drugs. Therefore, herein we evaluate seven flavonoids divided into three subclasses, isoflavone (genistein), flavone (apigenin and luteolin) and flavonol (fisetin, kaempferol, myricetin, and quercetin), for COVID-19 treatment using cell-based assays and in silico calculations validated with experimental enzymatic data. The flavonols were better SARS-CoV-2 inhibitors than isoflavone and flavones. The increasing number of hydroxyl groups in ring B of the flavonols kaempferol, quercetin, and myricetin decreased the 50% effective concentration (EC_50_) value due to their impact on the orientation of the compounds inside the target. Myricetin and fisetin appear to be preferred candidates; they are both anti-inflammatory (decreasing TNF-α levels) and inhibit SARS-CoV-2 mainly by targeting the processability of the main protease (M^pro^) in a non-competitive manner, with a potency comparable to the repurposed drug atazanavir. However, fisetin and myricetin might also be considered hits that are amenable to synthetic modification to improve their anti-SARS-CoV-2 profile by inhibiting not only M^pro^, but also the 3′–5′ exonuclease (ExoN).

## 1. Introduction

Coronavirus disease 2019 (COVID-19) is the most recent global pandemic of the 21st century, causing over 530 million confirmed cases and 6.29 million deaths worldwide from December 2020 until May 2022 [1]. Despite the fast development of vaccines and approved drugs by the U.S. Food and Drug Administration (FDA), e.g., remdesivir and baricitinib [2], severe acute respiratory syndrome coronavirus 2 (SARS-CoV-2) is still circulating due to variants of concern (VoC) that escape the humoral immune response; antiviral resistance might occur in the future [3,4,5,6]. Therefore, drug development against COVID-19 is still urgently needed in the current pandemic.

Basically, COVID-19 pathophysiology begins with SARS-CoV-2 virus binding to a type 1 transmembrane protein, known as angiotensin-converting enzyme 2 (ACE2), found on the host cell’s membrane. Upon viral fusion and release of the viral genome, several viral enzymes are critical in the replication process: e.g., the main protease (M^pro^) and papain-like protease (PL^pro^), which are responsible for polyprotein chain cleavage; the RNA-dependent RNA polymerase (RdRp), which replicates and transcribes the viral genome; and the 3′–5′ exonuclease (ExoN), which serves a proofreading function to maintain the integrity of the virus genome [7,8]. These enzymes were widely evaluated as feasible targets for drug repurposing, the design of novel drugs, and natural products screening [9,10,11,12].

Despite the great interest in drug repurposing to accelerate the identification of safe and effective compounds in humans that can cure or prevent COVID-19, there are some limitations based on their pharmacokinetic profile and required clinical concentration [13]. Natural products, due to their high safety profile, suitable bioavailability, and low level of adverse effects compared to synthetic drugs, are considered good candidates as novel antivirals, or as scaffolds for semisynthetic drugs [14]. In this regard, a class of natural polyphenols known as flavonoids, found in different amounts in plants, is being considered for the treatment of different diseases [15,16,17]. Flavonoids gained particular attention as potential anti-SARS-CoV-2 agents due to their ability to target some essential viral proteins in their entry and replication steps [17,18,19,20]. However, most of these reports are based on in silico screening or viral growth assessed in Vero-E6- and A549-cells, which are not considered ideal cellular models for SARS-CoV-2 infection [21,22]; thus, a better characterization of flavonoids for COVID-19 is still needed. Additionally, since infection by SARS-CoV-2 is associated with leukopenia and uncontrolled release of pro-inflammatory cytokine mediators in critically ill patients [23], and as previous reports for other infectious diseases demonstrated the immunomodulatory and anti-inflammatory effects of flavonoids [24], it is reasonable to expect that this class of natural products has a multi-target activity against COVID-19. Recently, this hypothesis was reinforced by Alzaabi and coworkers, who identified luteolin (a flavone) and quercetin (a flavonol) as promising multi-target compounds against SARS-CoV-2 that decrease both viral replication and pro-inflammatory mediators’ levels [25].

**Figure 1:**
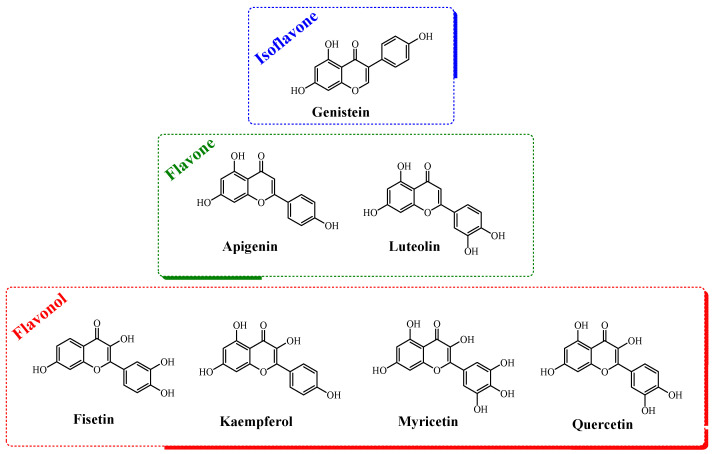
The chemical structures of the flavonoids evaluated in this work.

Based on the importance of natural products in the development of drugs against COVID-19 and the critical participation of certain viral enzymes in the replication process of the pathogen, e.g., exonucleases and proteases, the present work reports the screening of seven flavonoids (divided into isoflavone, flavones, and flavonols, Figure 1) using Calu-3-based infectivity assays (a type II pneumocyte line considered physiologically relevant in SARS-CoV-2 infection). The corresponding anti-inflammatory profile of each flavonoid was also determined in Calu-3 cells, as assessed by the levels of interleukin-6 (IL-6) and tumor necrosis factor-alpha (TNF-α). Finally, to identify the possible targets of the assayed compounds, in silico calculations regarding their binding capacity for the SARS-CoV-2 spike, RdRp, ExoN, PL^pro^, and M^pro^ proteins were carried out and correlated with the experimental enzymatic data for SARS-CoV-2 ExoN and M^pro^.

## 2. Materials and Methods

### 2.1. General Materials

Genistein, apigenin, luteolin, fisetin, kaempferol, myricetin, quercetin, remdesivir (RDV), carboxymethyl cellulose (CMC), tris(4-(dimethylamino)phenyl)methylium chloride (crystal violet), formaldehyde phosphate buffer solution (PBS), ethanol, dimethyl sulfoxide (DMSO), sulfuric acid (H_2_SO_4_), and 3,3’,5,5’-tetramethylbenzidine (TMB) were purchased from Sigma-Aldrich/Merck (St. Louis, MO, USA).

### 2.2. Cells and Virus

African green monkey kidney (Vero, subtype E6) and human lung epithelial (Calu-3) cells were cultured in high-glucose Dulbecco’s modified Eagle medium (DMEM—HyClone, Logan, Utah) supplemented with 100 U/mL penicillin, 100 μg/mL streptomycin (P/S—Thermo Fisher Scientific^®^, Waltham, MA, USA), and 10% fetal bovine serum (FBS—HyClone, Logan, UT, USA), and then incubated at 37 °C with 5% carbon dioxide (CO_2_).

The SARS-CoV-2 B.1 lineage (GenBank #MT710714) was isolated in Vero E6 cells from nasopharyngeal swabs of a confirmed case. All procedures related to virus culture were handled in the biosafety level 3 (BSL3) multiuser facility at *Fundação Oswaldo Cruz* (FIOCRUZ), Rio de Janeiro, Brazil, according to World Health Organization (WHO) guidelines [26].

### 2.3. Yield-Reduction Assays and Virus Titration

Calu-3 cells (2.0 × 10^5^ cells/well) in 96-well plates (Nalge Nunc Int’l, Rochester, NY, USA) were infected with a multiplicity of infection (MOI) of 0.1 for 1 h at 37 °C at 5% CO_2_. The inoculum was removed, and cells were incubated with different concentrations of genistein, apigenin, luteolin or fisetin (0.00, 0.63, 1.25, 2.50, 5.00, and 10.0 μM), kaempferol, myricetin, or quercetin (0.00, 0.10, 0.30, 1.00, 3.16, and 10.0 μM), and RDV (0.00, 0.0001, 0.001, 0.01, 0.10, 0.50, 1.0, 5.0, and 10.0 μM) in DMEM with 10% FBS. After 48 h, the virus content in the supernatant was quantified by plaque forming assays in Vero cells (2.0 × 10^4^ cells/well) according to our previous publications [10,27,28]. The virus titers were calculated by scoring for plaque-forming units (PFU/mL); and non-linear regression analysis of the dose–response curves were also performed to calculate the 50% effective concentration (EC_50_). All experiments were carried out at least three independent times, including a minimum of two technical replicates in each assay. Data were analyzed using Prism GraphPad software 8.0 (Windows GraphPad Software, San Diego, CA, USA). Values were presented as means ± standard deviations (SD).

### 2.4. Cytotoxic Assays

Vero cells (2.0 × 10^4^ cells/well) were treated for 3 days with different concentrations of flavonoids or RDV (ranging from 1 to 600 μM) following the procedure described previously [27]. Absorption was read at 595 nm using a plate spectrophotometer; the 50% cytotoxic concentration (CC_50_) was calculated using non-linear regression analysis from a dose–response curve. The assays were conducted in triplicate with results presented as means ± standard deviations (SD). The selectivity index (SI) for each assayed compound was calculated as the ratio of CC_50_ to EC_50_ values.

### 2.5. Measurements of Inflammatory Mediators

The levels of IL-6 and TNF-α were quantified from the supernatant of uninfected Calu-3 cells (MOCK), infected cells without treatment (NIL), and infected cells treated with flavonoid (10 µM) using specific kits following the manufacturer’s instructions (code #DY206 and #DY210 for IL-6 and TNF- α, respectively, from R&D Systems^®^ Inc., Minneapolis, MN, USA). Briefly, on the first day, the plate was sensitized with capture antibodies against the target cytokines and incubated overnight in a refrigerator. On the second day, the plate was washed three times with PBS containing 0.1% Tween, for later addition of blocking solution (PBS containing 0.1% bovine serum albumin) and incubation for 1 h. Then, supernatants from the viral replication inhibition assays in Calu-3 cells were added and incubated at room temperature for 2 h. After this, the plates were washed again and the detection antibody, streptavidin solution, and TMB were added. Upon observing the appearance of blue color, the reaction was stopped with 50 µL of 1 mol/L H_2_SO_4_, and absorption was measured in a spectrophotometer at a wavelength of 450 nm. The assays were conducted in triplicate, and IL-6 and TNF-α were quantified by a standard curve.

### 2.6. Molecular Docking Procedure

The chemical structures of the flavonoids apigenin, luteolin, genistein, fisetin, kaempferol, myricetin, and quercetin, as well as SARS-CoV-2 M^pro^ peptide substrate (CAS number 730985-86-1) and SARS-CoV-2 PL^pro^ peptide substrate (CAS number 167698-69-3), were built and energy-minimized using Density Functional Theory (DFT) through the Becke-3-Lee Yang Parr (B3LYP) method and the standard 6-31G* basis set, available in Spartan’18 software (Wavefunction, Inc., Irvine, USA). As the 3D structure for SARS-CoV-2 nsp-14 ExoN was not available, a structural model was built via the online Swiss Model software toolset (University of Basel, Basel, Switzerland) using the crystallographic structure of SARS-CoV nsp-14 as the template (Protein Data Bank (PDB) code: 5C8T) [29] and validated as previously described by Wang and co-workers [10]. In this study, two structural models were generated and validated: one considering the presence of one Mg(II) ion and the second with two Mg(II) ions. The positions of Mg(II) ions in the catalytic pocket of the models were identified according to the crystallographic structures for SARS-CoV ExoN (PDB code: 5C8T) [29] and the *N*-terminal ExoN domain of the epsilon subunit of *E. coli* DNA polymerase III (PDB code: 1J53) [30]. The structures for SARS-CoV-2 M^pro^, PL^pro^, RdRp, and spike proteins are available in the PDB with access codes 7K40, 6W9C, 7BV2, and 6VW1, respectively.

Molecular docking calculations were performed with GOLD 2020.2 software (Cambridge Crystallographic Data Centre, Cambridge, UK). Hydrogen atoms were added to the biomacromolecules according to the data inferred by software at pH 7.4. The standard function ChemPLP was used for each docking run. The molecular docking calculations were carried out in a select 8 Å radius spherical cavity around the active or allosteric binding site of each enzyme. Figures for the docking poses were generated using PyMOL Delano Scientific LLC software (Schrödinger, New York, NY, USA).

### 2.7. SARS-CoV-2 Exonuclease Reactions

The 3′-exonuclease, referred to as nsp14, and its protein cofactor, nsp10, were cloned and expressed based on the SARS-CoV-2 genome sequence. The SARS-CoV-2 ExoN nsp14/nsp10 complex was expressed and purified as described [10]. The RNA oligonucleotide (template-loop primer) was purchased from Dharmacon (Horizon Discovery, Lafayette, CO, USA).

The 3′ U-terminated template looped primer RNA (sequence shown in Section 3.4) was annealed by heating to 75 °C for 3 min and then cooling to room temperature in 1× ExoN reaction buffer. To a 14 μL solution of 71.4 nM ExoN complex (nsp14/nsp10) in 1× ExoN reaction buffer, 1 μL of DMSO with or without various concentrations of fisetin was added and incubated for 15 min at room temperature. Next, 5 μL of the annealed RNA (2 μM) in 1× ExoN reaction buffer was added to the ExoN/fisetin mixture and incubated at 37 °C for 15 min. The final concentrations of reagents in the 20 μL reactions were 50 nM nsp14/nsp10, 500 nM RNA, and 0 μM, 50 μM, or 150 μM fisetin in 5% DMSO. The 1× ExoN reaction buffer contains the following reagents: 40 mM Tris-HCl pH 8, 1.5 mM MgCl_2_, and 5 mM dithiothreitol (DTT). After incubation for 15 min, each reaction was quenched by adding 2.2 μL of an aqueous solution of ethylenediaminetetraacetic acid (EDTA, 100 mM). Following desalting using an Oligo Clean & Concentrator (Zymo Research Corporation, Irvine, CA, USA), the samples were subjected to Matrix-Assisted Laser Desorption/Ionization-Time Of Flight (MALDI-TOF) mass spectrometry (MS) analysis in a Bruker ultrafleXtreme^TM^ instrument (Bruker Daltonics, Billerica, MA, USA).

### 2.8. SARS-CoV-2 M^pro^ Inhibition

The capacity of fisetin and myricetin to inhibit the enzymatic velocity of SARS-CoV-2 M^pro^ was determined using a commercial kit (BPS Biosciences^®^, catalog number: #79955-1) following the procedure and recommendations from the literature and manufacturer [31,32]. This enzymatic kit is based on a FRET assay using a substrate peptide of M^pro^ labeled with a fluorescent dye (Dabcyl) and an acceptor−quencher (Edans) at the *N*- and *C*-terminus, respectively. The substrate peptide does not fluoresce in the uncleaved state, where the quencher blocks the fluorescence of the dye. However, after M^pro^ cleaves the substrate, the fluorescence of the dye is de-quenched, and an emission signal is observed. An inhibitor blocking the activity of M^pro^ will prevent FRET-peptide cleavage and a reduced fluorescence signal will be observed. Briefly, 88.8 nM M^pro^ was incubated overnight in reaction buffer (20 mM Tris pH 7.3, 100 mM NaCl, 1 mM EDTA, 1 mM DTT, and 1 µM BSA) containing 25 µM of substrate (modified peptide Dabcyl-KTSAVLQSGFRKME-Edans, CAS number 730985-86-1) and fisetin, myricetin, or GC376 (positive control) [33] at concentrations of 0.0, 0.08, 0.16, 0.31, 0.63, 1.25, 2.5, 5.0, and 10 µM. The fluorescence signal was measured at 460 nm upon excitation at 360 nm in a GloMax^®^ (Promega, Madison, WI, USA) plate reader. The Morrison’s inhibitory constant (*K_i_*) value was calculated by non-linear regression using GraphPad Prism 9. In addition to the inhibitory curve, an inhibitory screening of each flavonoid (10 μM) was also conducted under the same condition described above. A Michaelis–Menten plot was generated for 88.8 nM M^pro^ incubated overnight in assay buffer with substrate concentrations ranging from 0 to 100 µM in the presence and absence of 2.5 µM of fisetin or myricetin. After fluorescence quantification, the Michaelis–Menten constant (*K_m_*) and maximum velocity (*V_max_*) were also calculated by non-linear regression using GraphPad Prism 9 (Windows GraphPad Software, San Diego, CA, USA). Values were presented as means ± standard deviations (SD).

## 3. Results

### 3.1. Cell-Based Assays: SARS-CoV-2 Inhibition in Calu-3 Cells

**Figure 2:**
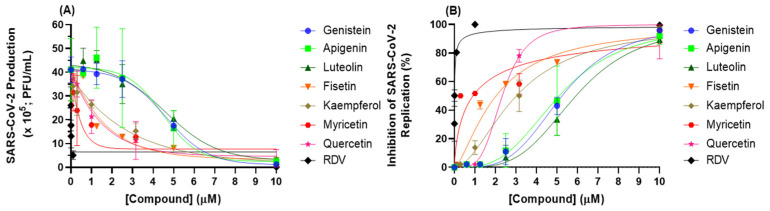
Antiviral activity of flavonoids and RDV (positive control). (**A**) SARS-CoV-2 production and (**B**) inhibition of SARS-CoV-2 replication in Calu-3 cells (densities of 2.0 × 10^5^ cells/well) infected with SARS-CoV-2 B.1 lineage at a MOI of 0.1.

Screening for the capacity of seven flavonoids (an isoflavone (genistein), flavones (apigenin and luteolin), and flavonols (fisetin, kaempferol, myricetin, and quercetin)) to inhibit SARS-CoV-2 replication was performed using cell-based assays. Figure 2 depicts the antiviral profile of each natural product; Table 1 summarizes the corresponding EC_50_, CC_50,_ and SI values. Flavonols showed lower EC_50_ values than isoflavone and flavones; among the flavonols, fisetin and myricetin presented the lowest EC_50_ values (2.03 ± 0.10 and 0.91 ± 0.05 μM, respectively), indicating that these two natural products are good candidates to inhibit SARS-CoV-2 replication. The CC_50_ value in most cases is approximately 60 times higher than the EC_50_ value, which provided a selective index (SI) consistent with an adequate safety profile in vitro, particularly for myricetin, with a SI value of 787 (Table 1).

### 3.2. Cell-Based Assays: Anti-Inflammatory Profile

**Figure 3:**
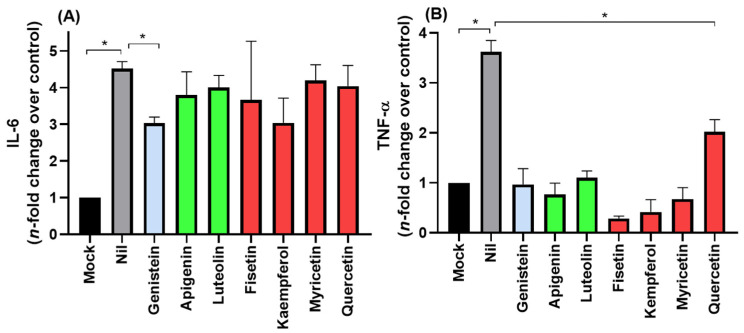
The anti-inflammatory profile of flavonoids was assessed using the (**A**) IL-6 and (**B**) TNF-α levels from uninfected Calu-3 cells supernatant (MOCK), SARS-CoV-2 infected cells without treatment (NIL), and infected and flavonoid treated cells with (10 µM). Blue, green, and red bars correspond to isoflavone, flavones, and flavonols, respectively. * *p* < 0.05.

COVID-19 frequently leads to fatal inflammatory responses and acute lung injury in critically ill patients. It was already reported that monocytes/macrophages from patients with severe COVID-19 may be the main source of uncontrolled levels of the pro-inflammatory mediators TNF-α and IL-6 in the peripheral blood of the respiratory tract [23]. Figure 3 depicts the flavonoids’ anti-inflammatory profile, focusing on TNF-α and IL-6 inhibition in Calu-3 SARS-CoV-2 infections. The isoflavone genistein was the only compound that reduced both IL-6 and TNF-α levels, while the other flavonoids impacted only TNF-α levels.

### 3.3. In Silico Calculations for the Main SARS-CoV-2 Targets to Natural Products

Experimental information about the mechanism of flavonoids regarding SARS-CoV-2 enzymes is lacking. Therefore, based on a literature survey of the proteins that play a major role in the pathogenicity of SARS-CoV-2, several target proteins were explored using molecular docking studies under different approximations (competitive and/or non-competitive interaction models). We performed this analysis on the spike glycoprotein with a single receptor binding domain in the presence and absence of ACE2 (PDB: 6VW1), due to its central role in viral entry [34], and the proteases PL^pro^ (PDB: 6W9C) and M^pro^ (PDB: 7K40), due to their role in cleavage and maturation of viral polyproteins [35]. Each protease was evaluated in silico in the absence and presence of the corresponding commercial synthetic peptide substrate (CAS numbers 730985-86-1 and 730985-86-1 for PL^pro^ and M^pro^, respectively), and for the two allosteric sites for M^pro^ [36,37]. Finally, RdRp and ExoN are critical for SARS-CoV-2 genome replication and proofreading, respectively [10]. Interestingly, these two last enzymes have Mg(II) ions in their catalytic pocket; since previous reports of the biological activity of flavonoids indicate that these natural products might interact with Mg(II) ions in inhibiting the integrase of the human immunodeficiency virus (HIV) [38,39,40], SARS-CoV-2 RdRp and ExoN were also considered feasible targets for in silico evaluation.

Table 2 summarizes the docking score values for all flavonoids evaluated in cell-based assays. In the GOLD 2020.2 software, a more positive score indicates better interactions; thus, docking score values suggest that all evaluated targets may feasibly interact with the flavonoids. However, the SARS-CoV-2 spike, RdRp, and PL^pro^ proteins had lower docking scores than SARS-CoV-2 ExoN and M^pro^, indicating that the latter two proteins might be the main targets for the evaluated natural products.

The replacement of one Mg(II) ion for two Mg(II) ions in the catalytic site of SARS-CoV-2 ExoN increased the binding capacity of all flavonoids. The flavonols (fisetin, kaempferol, myricetin, and quercetin) had a higher docking score than isoflavone (genistein) and the flavones (apigenin and luteolin); while among the flavonols, fisetin and myricetin appear to be the best candidates for SARS-CoV-2 nsp14 ExoN inhibition.

The interaction between flavonoids and SARS-CoV-2 M^pro^ had a better fit in the presence of substrate than in its absence, suggesting a non-competitive inhibitory mechanism. Among all flavonoids, fisetin and myricetin had the highest docking scores, indicating that M^pro^ might also be a feasible target for these compounds.

### 3.4. In Silico Evaluation for SARS-CoV-2 nsp-14 ExoN and Enzymatic Validation 

**Figure 4:**
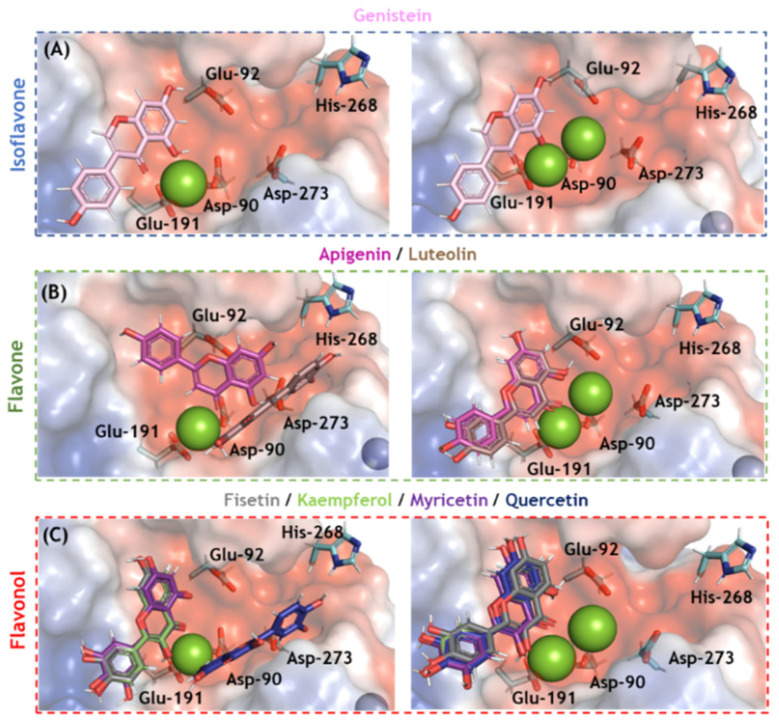
The electrostatic potential map (−68.065 and +68.065 a.u, in red and blue, respectively) of the best docking poses for the flavonoids (**A**) isoflavone, (**B**) flavones, and (**C**) flavonols in the SARS-CoV-2 nsp14 ExoN active site in the presence of one and two Mg(II) ions (left and right, respectively). The catalytic amino acid residues, genistein, apigenin, luteolin, fisetin, kaempferol, myricetin, and quercetin are in stick representation in cyan, violet, pink, brown, gray, light green, purple, and marine, respectively. Mg(II) and Zn(II) ions are represented as green and indigo blue spheres, respectively. Hydrogen, oxygen, and nitrogen are shown in white, red, and dark blue, respectively.

**Figure 5:**
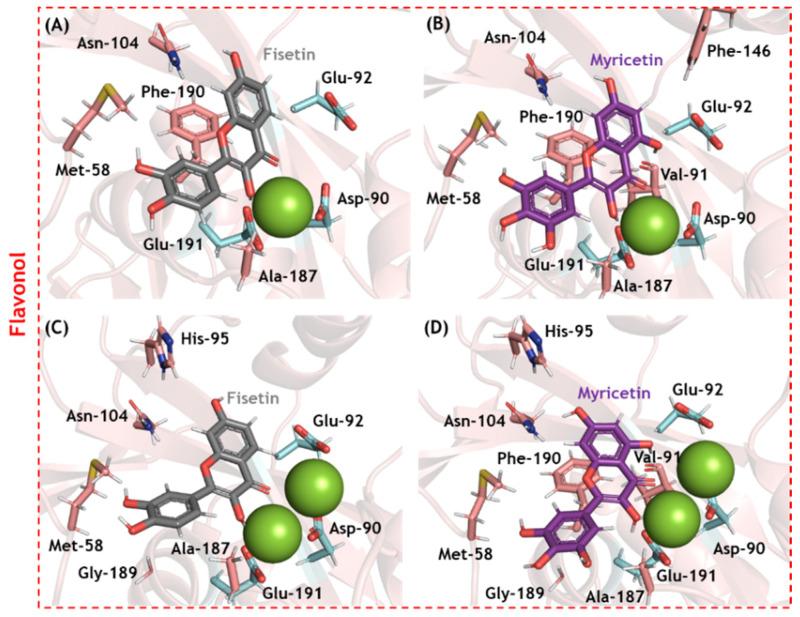
The main amino acid residues from SARS-CoV-2 nsp14 ExoN that interact with (**A**) fisetin and (**B**) myricetin in the presence of one Mg(II) ion, and (**C**) fisetin and (**D**) myricetin in the presence of two Mg(II) ions. The interactive and catalytic amino acid residues are in beige and cyan, respectively; fisetin and myricetin are in gray and purple, respectively. Mg(II) ions are represented as green spheres. Hydrogen, oxygen, and nitrogen are shown in white, red, and dark blue, respectively.

Molecular docking analysis was carried out for SARS-CoV-2 nsp14 ExoN in two different enzymatic 3D-structures differing in the number of cofactor Mg(II) ions in the catalytic pocket (Figure S1). Ten solutions for each flavonoid were obtained, and those poses with the best docking scores were subjected to more in-depth analysis. Figure 4 depicts the electrostatic potential maps for SARS-CoV-2 nsp14 ExoN in the presence of flavonoids with one or two Mg(II) ions. None of the flavonoids interacted with all the catalytic amino acid residues (Asp-90, Glu-92, Glu-191, His-268, and Asp-273); however, all potential inhibitors presented a favorable conformation to coordinate with Mg(II) ions, indicating that complexation with the cofactor might be the main mechanism by which flavonoids inhibit SARS-CoV-2 nsp14 ExoN, which is reminiscent of the integrase inhibitors of HIV [38,39,40]. Figure 5 shows the main amino acid residues that interact with fisetin and myricetin (the two flavonols with the highest docking scores). Table 2 and Table 3 summarize the interaction profiles. Molecular docking results indicate van der Waals and hydrogen bonding as the main intermolecular forces for the interaction between fisetin or myricetin and the amino acid residues in the SARS-CoV-2 nsp14 ExoN active site. Among the five catalytic amino acids, fisetin and myricetin interact with those closest to the Mg(II) ions: Asp-90, Glu-92, and Glu-191.

**Figure 6:**
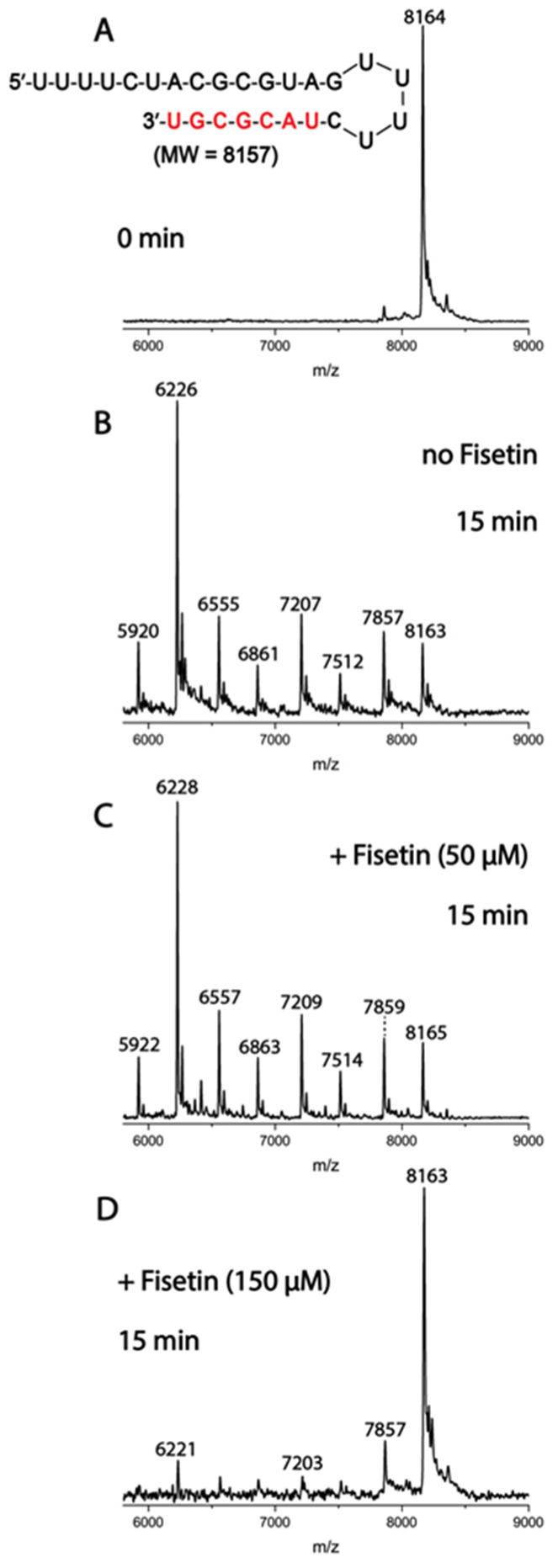
Inhibition of SARS-CoV-2 ExoN activity by fisetin. A mixture of 500 nM RNA (sequence shown at the top of the figure) and 50 nM SARS-CoV-2 pre-assembled exonuclease complex (nsp14/nsp10) was incubated in buffer solution at 37 °C for 15 min (**B**) in the absence and (**C**,**D**) presence of 50 μM and 150 μM fisetin. The (**A**) RNA and the (**B**–**D**) products of the ExoN reaction were analyzed by MALDI-TOF MS. The signal intensity was normalized to the highest peak. The peak at 8164 Da corresponds to the intact RNA (8157 Da expected).

To confirm the in silico trend for this target, enzymatic assays for processive cleavage by SARS-CoV-2 pre-assembled ExoN complex (nsp14/nsp10) of a specific ribonucleic acid (RNA) sequence were conducted in the presence and absence of fisetin. Molecular docking calculations suggested a very similar docking score value for fisetin and myricetin to ExoN. Therefore, enzymatic assays were conducted for both flavonols; however, the data are shown only for fisetin, as the results for myricetin were not clean and no inhibition was seen, even at 150 μM. Figure 6 depicts the mass spectra for the specific sequence of RNA without and with two different concentrations of fisetin. In the absence of fisetin, ExoN cleaves 1–7 nucleotides from the 3′-end of the RNA, while in the presence of 150 μM fisetin, ExoN activity was reduced, as shown by the reduced intensities of the fragmentation peaks and increased intact RNA peak. This profile indicates that fisetin might inhibit SARS-CoV-2 nsp14 ExoN at high concentration, in contrast to the EC_50_ values in the Calu-3 cell-based virus inhibition assays, suggesting that ExoN is probably not the main target for this natural product.

### 3.5. In Silico Evaluation for SARS-CoV-2 M^pro^ and Enzymatic Validation 

**Figure 7:**
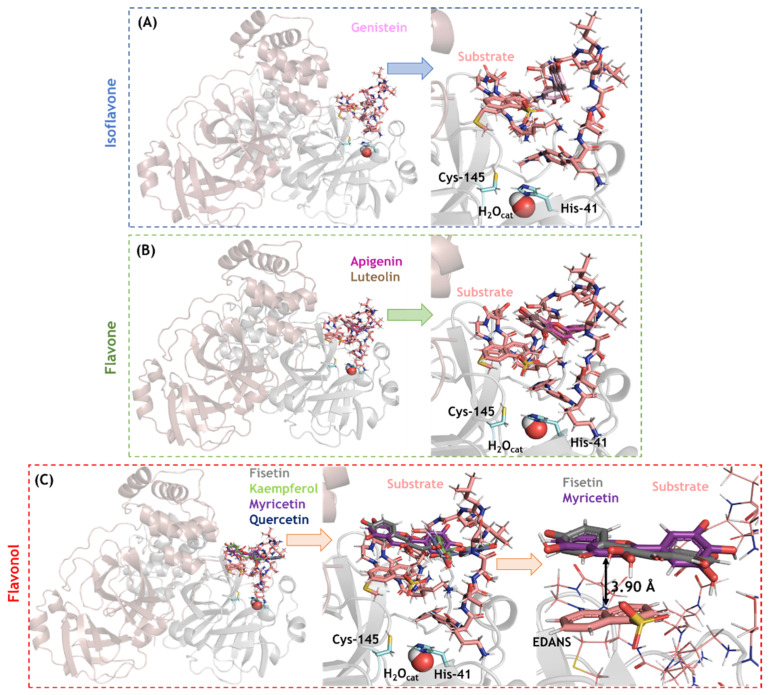
The 3D-representation of the best docking pose and the corresponding zoom representation for the flavonoids (**A**) isoflavone, (**B**) flavones, and (**C**) flavonols in the SARS-CoV-2 M^pro^ active site in the presence of peptide substrate (CAS number 730985-86-1). Catalytic amino acid residues genistein, apigenin, luteolin, fisetin, kaempferol, myricetin, and quercetin are in stick representation in cyan, violet, pink, brown, gray, light green, purple, and marine, respectively. Catalytic water (H_2_O_cat_) is shown in spherical configuration. Hydrogen, oxygen, and nitrogen are shown in white, red, and dark blue, respectively.

SARS-CoV-2 M^pro^ was another feasible target based on its molecular docking score; therefore, an in-depth analysis of isoflavone, flavones, and flavonols in the M^pro^/substrate complex system (which may help distinguish between a competitive and non-competitive inhibitory mechanism) was conducted. Ten solutions for each flavonoid were obtained, and those poses with the best docking scores were analyzed. In this case, all the flavonoids were docked inside the loop formed by the substrate in the active site. As depicted in Figure 7, genistein (isoflavone) is not completely buried within the substrate loop, while apigenin and luteolin (flavones) are buried more deeply in the substrate loop. The flavonols fisetin and myricetin showed the highest interactive profile, probably due to the presence of a hydrophobic interaction (π-stacking) between the aromatic ring of these flavonols with the acceptor−quencher (Edans) moiety of the substrate, within a distance of 3.90 Å (the zoom representation in Figure 7C).

**Figure 8:**
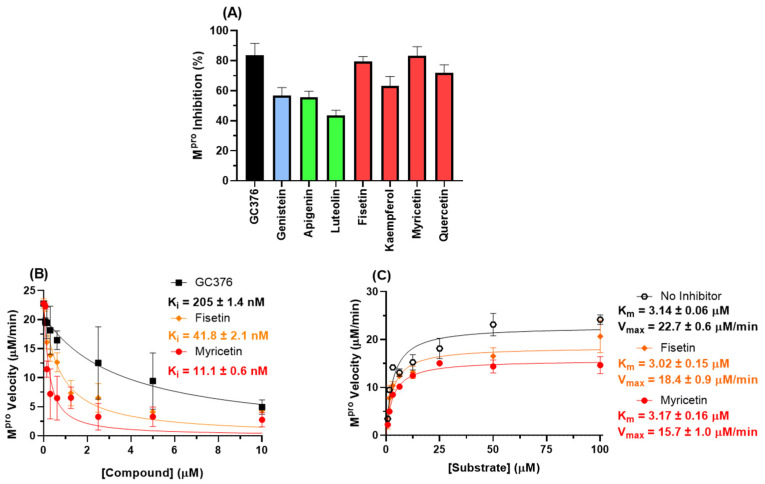
(**A**) Inhibition percentage of flavonoids (10 μM) to 88.8 nM M^pro^. Blue, green, and red bars correspond to isoflavone, flavones, and flavonols, respectively. (**B**) The enzymatic inhibition profile for fisetin, myricetin, and GC376 (positive control) for 88.8 nM M^pro^ in PBS. (**C**) Michaelis–Menten enzymatic mechanism for M^pro^ in the absence and presence of a fixed fisetin or myricetin concentration (2.5 µM) at different substrate concentrations.

To confirm the in silico trend for this target, experimental enzymatic screening was performed which indicated that fisetin and myricetin are the two main SARS-CoV-2 M^pro^ inhibitors (Figure 8A). Figure 8B,C show the enzymatic inhibition profile for fisetin, myricetin, and GC376 (positive control) and their corresponding enzymatic mechanisms. Morrison’s inhibitory constant (*K_i_*) value for the flavonols is lower than that for GC376, indicating that these natural products are good candidates to inhibit SARS-CoV-2 M^pro^. More specifically, myricetin has a *K_i_* value four times lower than fisetin, corroborating the experimental cell-based assays’ trend. The Michaelis–Menten constant (*K_m_*), in the absence and in the presence of flavonols, is the same within experimental error, while the maximum velocity (*V_max_*) value decreased in the presence of the flavonols, supporting a non-competitive inhibitory mechanism and validating the in silico approximation.

## 4. Discussion

Studies demonstrate the broad spectrum antiviral activity of flavonoids [18,19,20,41]. Indeed, these molecules can interfere in different virus infections, e.g., many aglycone and mono-glycoside flavonols, including fisetin, quercetin, rutin, and isoquercetin, are potent antivirals against influenza A and B viruses [41]. Recently, in silico calculations and Vero-based assays suggested this class of natural products as potential inhibitors of SARS-CoV-2, mainly by disturbing virus entry and protease activity [9,18,19,20,24,25]. However, there is little work validating these in silico calculations with enzymatic assays and confirming SARS-CoV-2 inhibition using physiologically relevant cells, making the information regarding the biological activity of flavonoids for COVID-19 treatment less convincing. Therefore, we performed pre-clinical study steps for isoflavone, flavones, and flavonols by screening for their antiviral and anti-inflammatory profile in Calu-3-based assays and evaluating their main targets using in silico calculations validated by enzymatic assays.

Using cell-based assays, we discovered that the tested flavonols (fisetin, kaempferol, myricetin, and quercetin) are more potent SARS-CoV-2 inhibitors than isoflavone (genistein) and flavones (apigenin and luteolin). In fact, kaempferol, quercetin, myricetin, fisetin, and their derivatives are the most documented flavonoids, with a broad spectrum of biological activities, and thus the best theoretical candidates as antivirals against SARS-CoV-2 [15]. Interestingly, our data indicate that the presence of more hydroxyl groups in ring B of the flavonols (kaempferol (one hydroxyl group), quercetin (two hydroxyl groups), and myricetin (three hydroxyl groups)) increased the capacity of these compounds to inhibit SARS-CoV-2 replication; i.e., they had EC_50_ values of 3.02 ± 0.15, 2.40 ± 0.12, and 0.91 ± 0.05 μM, respectively. This is probably due to their impact on the orientation of the compounds inside the enzyme target, which was supported by the experimental evidence in the inhibitory percentage of kaempferol, quercetin, and myricetin (10 μM) to SARS-CoV-2 M^pro^. The EC_50_ value for myricetin was comparable to that of atazanavir (ATV), a repurposed SARS-CoV-2 inhibitor that targets M^pro^ [27], reinforcing the good prospects of flavonols as SARS-CoV-2 antivirals. The CC_50_ value in most cases is about 60 times higher than the EC_50_ value, which provided a selective index (SI) consistent with an adequate safety profile.

All the natural products evaluated in this work might reduce the severity of COVID-19 symptoms by significantly decreasing the production of the pro-inflammatory mediator TNF-α. This finding corroborates previous studies that identified some flavonoids, e.g., fisetin, kaempferol, myricetin, astragalin, and rutin, as inhibitors of pro-inflammatory cytokines in other human infections [17,42]. An increase in TNF-α levels in the peripheral blood of the respiratory tract is associated with patients with severe COVID-19 [23]. Our data suggest that flavonols, mainly fisetin and myricetin (all natural products), engage in multi-target activity against COVID-19.

In silico calculations for different SARS-CoV-2 proteins (spike, RdRp, ExoN, PL^pro^, and M^pro^) were carried out to identify flavonoids’ main targets; molecular docking score values clearly suggest SARS-CoV-2 ExoN and M^pro^ as feasible targets. The two main in vitro SARS-CoV-2 inhibitors, fisetin and myricetin, had the highest docking scores when compared with the other flavonoids, providing confiability for the in silico approach.

Generally, the SARS-CoV-2 ExoN has two Mg(II) ions in the catalytic site; however, the absence of the second Mg(II) ion was already reported and attributed to the lack of substrate or product binding [29,30,43]. For this reason, two possibilities were evaluated in our in silico models. We found that the presence of two Mg(II) ions improved the docking score and binding profile of fisetin and myricetin, suggesting that these natural products might interact with ExoN in the presence of substrate or product binding, similar to clinically approved HIV integrase inhibitors (raltegravir, elvitegravir, dolutegravir, bictegravir, and cabotegravir) [38,39,40]. Experimental enzymatic validation showed that fisetin can interfere with RNA processing by ExoN with a 74-fold lower potency than the EC_50_ value; thus SARS-CoV-2 ExoN is likely not the main target for flavonols.

On the other hand, fisetin and myricetin non-competitively inhibited SARS-CoV-2 M^pro^ processability with 48- and 82-fold higher potency (respectively) than their corresponding EC_50_ value, suggesting M^pro^ as the main target for flavonols. Indeed, the docking score value for M^pro^ in the protease/substrate complex was slightly higher than for ExoN; and the impact of an increasing number of hydroxyl groups in ring B of the flavonols was identified for M^pro^ more clearly than for ExoN (myricetin’s pose in the protease’s substrate loop is totally different compared with those of kaempferol and quercetin), reinforcing the claim that this protease is the main target.

Overall, our results reveal that the flavonols myricetin and fisetin are the best candidates for further testing, given both their anti-inflammatory effects and their inhibition of SARS-CoV-2 replication mainly by targeting M^pro^ processability in a non-competitive fashion (interaction with the protease/substrate complex in the active site), which demonstrates a potency comparable to the repurposed drug ATV. However, considering the design and structure of clinically approved HIV-integrase inhibitors, fisetin and myricetin might also be considered hits that will be advanced further into the development of lead compounds. For instance, their anti-SARS-CoV-2 activity profile of targeting both M^pro^ and ExoN might be improved by inserting hydrophobic moieties into rings A or B to better orient the ligands in the ExoN hydrophobic cleft, increasing the inhibitors’ affinity for Mg(II) ions, thereby reducing ExoN’s ability to proofread RNA. Approximately 19% of FDA-approved drugs on the market are semisynthetic [14,44]; this bolsters our positive opinion regarding the continuous effort to identify natural products as a hit for future development of leads based on their phytochemical core.

Moreover, we understand that while most natural products are still not considered in clinical studies, mainly due to the lack of abundant natural supply or the exhaustive laboratory cost to synthesize them, we consider that there is enough world capital to invest in the synthesis of novel drugs. However, investment is proportional to financial return; the pharmaceutical industry is betting on molecules with a much greater degree of complexity due to the inherent barriers that prevent competitors from synthesizing generics, e.g., PF-07321332 in PAXLOVID^TM^, a SARS-CoV-2 M^pro^ inhibitor [45]. In this regard, the straightforward chemical accessibility of flavonoids facilitates drug discovery and optimization mainly by health agencies focusing on neglected populations. We hope that COVID-19 will change this paradigm, and that flavonoids’ economic accessibility encourages collaboration with poor populations to produce certain plants that might be used for the extraction of compounds of interest, thus helping achieve the third and eighth Sustainable Development Goals (SDGs) of the United Nations [46].

## Figures and Tables

**Table 1:** The 50% effective concentration (EC_50_ for MOI 0.1), 50% cytotoxic concentration (CC_50_), and selectivity index (SI) for the flavonoids and RDV (positive control) in Calu-3 cells.

Compound	EC_50_ (μM)	CC_50_ (μM)	SI
Genistein	5.25 ± 0.26	305 ± 15	58.1
Apigenin	5.11 ± 0.26	302 ± 15	59.1
Luteolin	5.92 ± 0.30	332 ± 17	56.1
Fisetin	2.03 ± 0.10	256 ± 13	126
Kaempferol	3.02 ± 0.15	357 ± 18	118
Myricetin	0.91 ± 0.05	716 ± 36	787
Quercetin	2.40 ± 0.12	852 ± 43	355
RDV	0.0305 ± 0.0031	512 ± 30	1.68 × 10^4^

**Table 2:** Docking score values (dimensionless) for the flavonoids in spike, RdRp, ExoN, PL^pro^, and M^pro^.

	Spike		RdRp		ExoN		PL^pro^		M^pro^			
Compound	Without ACE2	With ACE2	Without RNA	With RNA	1 × Mg(II)	2 × Mg(II)	Without Substrate	With Substrate	Without Substrate	With Substrate	Allosteric Site 1	Allosteric Site 2
Genistein	39.9	39.6	37.3	36.6	45.7	46.3	46.1	47.3	52.6	53.3	50.5	47.7
Apigenin	38.8	42.3	41.2	43.9	44.9	49.9	46.8	48.0	50.9	54.8	45.0	44.8
Luteolin	42.3	43.9	43.2	42.5	48.9	53.4	48.5	48.9	53.2	56.0	48.1	48.3
Fisetin	42.9	42.0	47.0	46.3	53.7	56.1	50.4	50.9	55.2	56.9	50.7	50.4
Kaempferol	38.4	07.6	42.2	37.1	54.9	55.8	48.9	49.5	51.5	52.1	48.0	46.4
Myricetin	43.0	28.3	48.4	47.1	55.2	57.9	47.7	48.9	56.8	64.0	52.8	53.2
Quercetin	44.7	34.8	46.4	45.9	46.5	51.5	50.5	50.7	51.8	55.7	51.1	50.2

**Table 3:** The main amino acid residues that interact with fisetin and myricetin in the catalytic pocket of SARS-CoV-2 nsp14 ExoN in the presence of one or two Mg(II) ions.

Compound	Amino Acid Residue	Interaction	Distance (Å)
Fisetin (1 × Mg(II))	Met-58	Van der Waals	3.40
	Glu-92	Van der Waals	3.10
	Asn-104	Hydrogen bonding	2.80
	Phe-146	Van der Waals	3.80
	Ala-187	Van der Waals	2.00
	Phe-190	Van der Waals	3.40
	Glu-191	Hydrogen bonding	2.10
Myricetin (1 × Mg(II))	Met-58	Van der Waals	2.30
	Asp-90	Van der Waals	3.80
	Val-91 (C=O)	Hydrogen bonding	1.70
	Glu-92	Van der Waals	2.80
	Asn-104	Hydrogen bonding	3.30
	Phe-146	Van der Waals	3.40
	Ala-187	Van der Waals	2.20
	Phe-190	Van der Waals	3.80
	Glu-191	Hydrogen bonding	2.10
Fisetin (2 × Mg(II))	Met-58	Van der Waals	2.30
	Asp-90	Van der Waals	3.70
	Glu-92	Van der Waals	2.40
	His-95	Van der Waals	2.90
	Asn-104	Van der Waals	2.80
	Ala-187	Van der Waals	3.40
	Gly-189	Van der Waals	2.80
	Glu-191	Hydrogen bonding	2.20
Myricetin (2 × Mg(II))	Met-58	Van der Waals	2.60
	Asp-90	Van der Waals	3.80
	Val-91 (C=O)	Hydrogen bonding	1.70
	Glu-92	Van der Waals	3.00
	His-95	Van der Waals	2.80
	Asn-104	Hydrogen bonding	2.20
	Ala-187	Van der Waals	2.40
	Gly-189	Van der Waals	3.00
	Phe-190	Van der Waals	3.70
	Glu-191	Hydrogen bonding	2.90

## Data Availability

All analyzed data are contained in the main text and in the Supplementary Material of the article. Raw data are available from the authors upon request.

## Supplementary Materials

The following supporting information can be downloaded at: https://www.mdpi.com/article/10.3390/v14071458/s1, Figure S1: Superposition of the SARS-CoV nsp14 (PDB code: 5C8T, in gray); (A) SARS-CoV-2 nsp14 model 1 (with one Mg(II) ion) and (B) SARS-CoV-2 model 2 (with two Mg(II) ions). The beige and orange colors are ExoN and N7-MTase domains, respectively. Selected amino acid residues in the ExoN catalytic site are represented using stick representations in cyan. The cosubstrate involved in methyltransferase, *S*-adenosyl methionine (SAM), is represented using a stick model in green. Hydrogen atoms were omitted for better clarity. The oxygen (red), nitrogen (dark blue) and sulfur (yellow) atoms are presented in the stick structure. The Mg(II) (dark green) and Zn(II) (indigo blue) ions are represented as spheres.
