# Supplementary material for: Commercially Available Flavonols Are Better SARS-CoV-2 Inhibitors than Isoflavone and Flavones
Source: Viruses. 2022 Jun 30;14(7):1458. doi: 10.3390/v14071458 (PMC9324382; doi:10.3390/v14071458)
Supplement: Supplementary file 1 [file viruses-14-01458-s001.zip › viruses-1788261-supplementary.pdf]

SUPPLEMENTARY MATERIAL

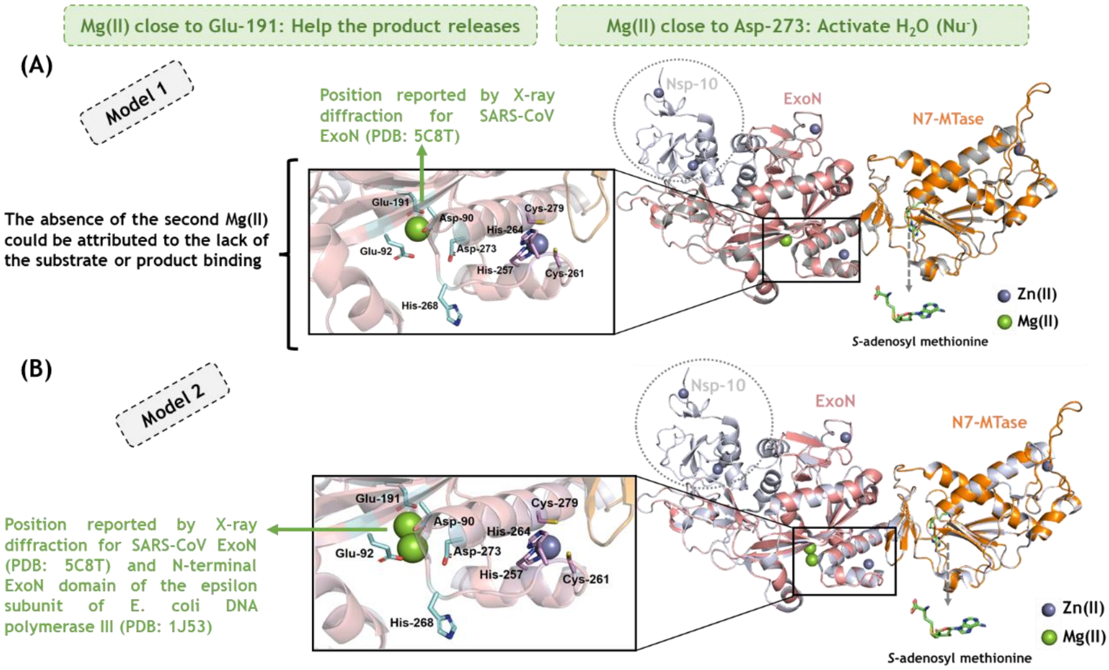

Figure S1. Superposition of the SARS-CoV nsp14 (PDB code: 5C8T, in gray); (**A**) SARS-CoV-2 nsp14 model 1 (with one Mg(II) ion) and (**B**) SARS-CoV-2 model 2 (with two Mg(II) ions). The beige and orange colors are ExoN and N7-MTase domains, respectively. Selected amino acid residues in the ExoN catalytic site are represented using stick representations in cyan. The cosubstrate involved in methyltransferase, S-adenosyl methionine (SAM), is represented using a stick model in green. Hydrogen atoms were omitted for better clarity. The oxygen (red), nitrogen (dark blue) and sulfur (yellow) atoms are presented in the stick structure. The Mg(II) (dark green) and Zn(II) (indigo blue) ions are represented as spheres.
